# Application of MODIFI to the adaptation of a complex, multilevel intervention to enhance access to high-quality cancer services in rural cancer hospitals

**DOI:** 10.1186/s43058-025-00805-x

**Published:** 2025-10-16

**Authors:** Mary C. Schroeder, Sarah A. Birken, Ingrid M. Lizarraga, M. Alexis Kirk, Cheyenne R. Wagi, Jacklyn M. Engelbart, Erin C. Johnson, Madison M. Wahlen, Aaron T. Seaman, Mary E. Charlton

**Affiliations:** 1 Division of Health Services Research, University of Iowa College of Pharmacy, 180 South Grand Ave, 346 CPB , Iowa City, IA 52242 USA; 2 Department of Implementation Science, Wake Forest University School of Medicine, 1 Medical Center Blvd, Winston-Salem, NC 27157 USA; 3 Department of Surgery, University of Iowa Roy J. and Lucille A. Carver College of Medicine, 200 Hawkins Drive, 4636 JCP, Iowa City, IA 52242 USA; 4 Independent Consultant, Apex, NC USA; 5 Department of Surgery, University of Iowa Roy J. and Lucille A. Carver College of Medicine, 200 Hawkins Drive, 1527 JCP, Iowa City, IA 52242 USA; 6 Department of Management and Entrepreneurship, Tippie College of Business, University of Iowa, 21 East Market Street, W250 PBP, Iowa City, IA 52242 USA; 7 Department of Epidemiology, University of Iowa College of Public Health, 145 N. Riverside Drive, Iowa City, IA 52242 USA; 8 Department of Internal Medicine, University of Iowa Roy J. and Lucille A. Carver College of Medicine, 200 Hawkins Drive, SE631 GH, Iowa City, IA 52242 USA; 9 Department of Epidemiology, University of Iowa College of Public Health, 145 N. Riverside Drive, S453 CPHB, Iowa City, IA 52242 USA

**Keywords:** Adaptation methods, Core functions, Evidence-based intervention, Focus groups, Interviews, Cancer, Rural hospital

## Abstract

**Background:**

The University of Kentucky Markey Cancer Center Affiliate Network (MCCAN) is a complex, multilevel evidence-based intervention (EBI) aimed at enhancing access to high-quality cancer services for under-served patients. MCCAN is promising but has not been scaled beyond its original context. We aimed to adapt MCCAN, originally developed in Kentucky, to address systematic differences that threatened its implementation and effectiveness in a new context, Iowa, yielding the Iowa Cancer Affiliate Network (I-CAN).

**Methods:**

We report our adaptation of MCCAN using the Making Optimal Decisions for Intervention Flexibility during Implementation (MODIFI) approach: (1) identify key information about MCCAN, learning about Kentucky and Iowa contexts and users; (2) adapt MCCAN’s forms while leaving its core functions intact to produce I-CAN; and (3) evaluate I-CAN. Specifically, we conducted studies to identify MCCAN’s forms and core functions, gathered extensive knowledge of the original and new contexts, and identified systematic differences between the two. We created a matrix to map MCCAN’s core functions to its original forms, contextual differences between Kentucky and Iowa, and proposed adapted forms to produce I-CAN. We interviewed I-CAN affiliates to assess perceptions of acceptability, feasibility, and efficacy.

**Results:**

MCCAN forms were mapped to eight intervention and 10 implementation core functions. Adaptation was required for 11 core functions, as contextual differences impacted the ability of the original forms of those core functions to be carried out in the new context. Contextual differences reflected existing relationships and referral patterns, as well as available resources (e.g., personnel and infrastructure). Lack of familiarity with the intervention process and outcomes limited the ability of I-CAN affiliates to evaluate potential adapted forms. Forms evolved as I-CAN affiliates gained practical experience in applying them and/or experienced changes in organizational structure, personnel, etc.

**Conclusions:**

We successfully adapted MCCAN, a complex, multilevel EBI designed to support community hospitals and enhance access to high-quality cancer services and programs in Kentucky to improve care for patients in Iowa affected by cancer—nearly half of whom reside in rural areas. Our application of MODIFI suggests several opportunities for refinement to advance successful EBI adaptation.

**Trial registration:**

ClinicalTrials.gov, NCT05645328. Registered 01 December 2022, https://clinicaltrials.gov/study/NCT05645328

**Supplementary Information:**

The online version contains supplementary material available at 10.1186/s43058-025-00805-x.

Contributions to the literature

- The Markey Cancer Center Affiliate Network (MCCAN) was found to improve guideline-concordant cancer care. Scaling up evidence-based interventions (EBIs) such as MCCAN as broadly as is feasible and appropriate can have great public health impact. We adapted MCCAN to scale its benefits to patients in rural Iowa.
- We reported our adaptation of MCCAN using MODIFI, representing the first empirical application of the approach of which we are aware.
- Applying MODIFI identified several opportunities for refinement, including identifying EBI core functions prior to collecting context and user data, anchoring adaptations on differences between original and new contexts, and mapping adapted forms onto core functions.

## Background

Hospitals in rural areas face significant barriers in delivering high-quality cancer care due to physical isolation, challenges in recruiting and retaining specialists, limited resources and services, and thin profit margins [1–5]. These barriers have contributed to lower rates of screening, later stage presentation, lower enrollment in clinical trials, poorer-quality or less-advanced diagnostics and treatment, greater fragmentation of care, and higher cancer mortality in rural cancer patients [4–8]. In response to the disparities in access and outcomes observed in their own state, the University of Kentucky Markey Cancer Center developed the Markey Cancer Center Affiliate Network (MCCAN) with a goal of enhancing access to high-quality cancer services and programs for cancer patients treated in community hospitals throughout Kentucky. Specifically, MCCAN provides affiliate hospitals educational and training resources, access to clinical trials and specialized services, and support in achieving and maintaining accreditation by the American College of Surgeons Commission on Cancer (CoC). The CoC accredits more than 1500 US hospitals that demonstrate achieving regularly revised quality standards intended to promote cancer prevention, research, education, and monitoring of comprehensive quality care to improve the survival and quality of life for patients with cancer [9–12]. As a network, MCCAN has been shown to be an effective, evidence-based intervention (EBI); compared to matched control hospitals, MCCAN affiliates were more likely to deliver guideline-concordant cancer care and achieve CoC accreditation [13].

Expanding the use of EBIs, scaling-up and scaling-out, as broadly as is feasible and appropriate can have great public health impact [14]. After learning about MCCAN, our team applied for and was awarded an R01 grant from the National Cancer Institute (NCI) to conduct a hybrid effectiveness-implementation study, adapting MCCAN for a new context and establishing the Iowa Cancer Affiliate Network (I-CAN) for rural hospitals in Iowa. EBIs are often adapted post hoc in reaction to poor fit [15], resulting in implementation strategies that potentially deviate from intervention core functions and compromise effectiveness [16]. In contrast, I-CAN was to be designed a priori in a systematic, proactive process of study and adaptation of MCCAN. Specifically, our grant application proposed to use Kirk et al.’s Model for Adaptation Design and Impact (MADI) framework for adaptation [17], first identifying EBI core functions and forms as conceptualized by Perez et al. [18].

Principles from Perez et al. and Kirk et al. were synthesized in the recently published Making Optimal Decisions for Intervention Flexibility during Implementation (MODIFI) approach [19]. Thus, we report our adaptation effort using MODIFI, representing the first published study of which we are aware to apply MODIFI to an empirical study, aside from the case study featured in the paper in which MODIFI was introduced.

## Methods

**Fig. 1:**
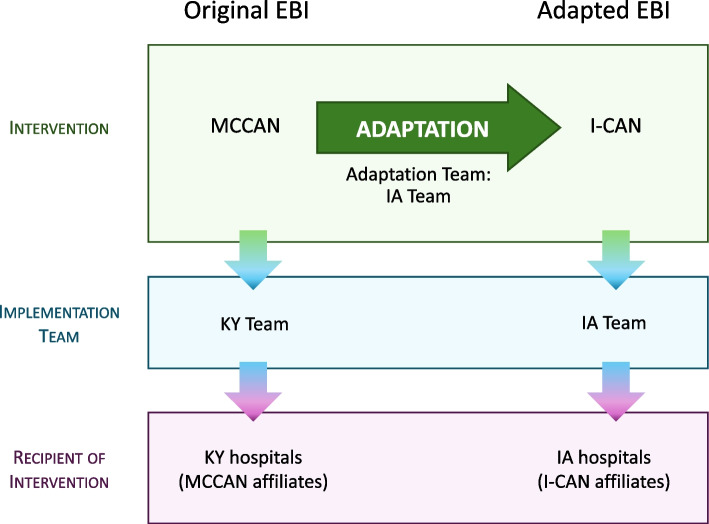
Schematic of original and adapted evidence-based intervention (EBI) and key partners. Abbreviations: MCCAN = Markey Cancer Center Affiliate Network, I-CAN = Iowa Cancer Affiliate Network, KY = Kentucky, IA = Iowa

We used the Making Optimal Decisions for Intervention Flexibility during Implementation (MODIFI) approach to adapt MCCAN and establish I-CAN for implementation in Iowa. MODIFI identifies three prerequisites for adaptation: selection of intervention, need for adaptation, and adaptation team. In our project, MCCAN was the selected EBI and the lack of any cancer care network in Iowa (new context) served as the need for adaptation. From inception, the adaptation team included primary users who implement the adapted EBI (I-CAN team members, study investigators, authors of this paper), individuals with expertise in the original EBI (MCCAN team, study investigators), and individuals with expertise in implementation science generally and intervention adaptation methods specifically (I-CAN team members, study investigators, authors of this paper). The members of our I-CAN team, who would eventually implement the adapted EBI, led the study of the original EBI. Figure 1 provides a schematic of the roles and relationships between the key partner for the original and adapted EBI.

After these prerequisites, MODIFI involves three steps: (1) identifying key information about the EBI, learning about the local context, and learning about the users; (2) adapting the EBI’s forms while leaving the core functions intact; and (3) evaluating whether the EBI adaptation works for the identified users within the local context. The Consolidated Criteria for Reporting Qualitative Research (COREQ) checklist [20] were used for reporting (see Additional file 1).

### MODIFI step 1

#### Identify key information about the original EBI and context

MADI, on which MODIFI was based, recommends first identifying the EBI’s core functions (i.e., EBI features that drive effectiveness and therefore must be retained to ensure effectiveness in new contexts). Kirk et al.’s core functions identification methods are described in detail elsewhere and have been applied in several peer-reviewed studies [16, 21, 22]. We used an inductive approach to identify MCCAN’s forms and core functions, as the network had not been previously evaluated or defined, and to ensure that the full universe of forms and core functions would be identified. We reported methods for identifying MCCAN core functions and our findings in a previously published study [23]. Briefly, conducted semi-structured interviews with MCCAN partners and affiliates. From interview transcripts, we identified themes regarding how MCCAN engaged affiliates in improving care quality (intervention forms) and implementing CoC standards (implementation forms). The identified forms were then categorized into the following domains, consistent with MADI [17], and distilled into core functions based on an underlying theory of change: who/where (the people and places essential to implementation and the purpose and function of each place or role involved in the intervention), what (the aspects of the intervention that were essential to its success), why (the purpose and goals of the intervention), and how (the methods and conditions necessary to successfully implement the intervention).

We conducted an additional study to understand the value of network activities and CoC accreditation from the perspective of the MCCAN affiliates (recipients of the original EBI). We conducted semi-structured interviews with MCCAN affiliate hospital clinicians and administrators to identify (1) facilitators and barriers to joining the network and/or obtaining CoC accreditation, and (2) MCCAN services and activities perceived to be most helpful, effective, or valuable for CoC accreditation [24].

Finally, we participated in biweekly meetings with the MCCAN team and affiliates to gain deeper understanding of MCCAN and to learn from their experience implementing the intervention. These meetings involved conceptual and logistical discussions about MCCAN recruitment and engagement, resources and services provided to affiliates, and evaluation of affiliates progress toward CoC accreditation and support towards their achievement of these standards. Detailed notes were taken during these meetings and/or recorded. MCCAN also provided I-CAN with materials such as resources for recruitment and onboarding; checklists with each accreditation standard and ideas for how to meet them; and templates of timelines and Gantt charts.

#### Identify key information for the new EBI and context

We selected members of the I-CAN team to optimize the adaptation and implementation process. Individuals were chosen for their extensive knowledge of and experience with rural health services, cancer care delivery and infrastructure, CoC accreditation, and data collection and quality improvement efforts in Iowa. Team members served in the various roles relevant to the users and recipients of the adapted EBI: CoC State Chair, CoC Cancer Liaison Physician, community cancer center director, Director and Principal Investigator of the Iowa Cancer Registry, co-leader of the Cancer Epidemiology and Population Science Program of the University of Iowa Holden Comprehensive Cancer Center, and breast surgical oncologist with experience practicing as a general surgeon in a rural hospital.

Additionally, we (the I-CAN team) conducted qualitative studies to more broadly understand the strengths and challenges of cancer care provision by rural providers, as well as the dynamics of referral pathways and physician collaboration across healthcare systems [25, 26]. During the first two years of the grant, while studying MCCAN, we also conducted multiple site visits, touring the rural I-CAN affiliate hospitals and meeting key partners. Goals for the initial site visit were to (1) assess current oncology services and programs, (2) clarify cancer program goals and vision for the future, (3) identify any current accreditations and/or certifications and discuss aspirations and timelines for future accreditations and/or certifications, and (4) identify potential areas of support that could be provided by I-CAN. During this visit, we provided an overview of the grant objectives and a presentation on the value of CoC accreditation. Baseline data were collected through surveys and gap analyses conducted with key partners (administrators, providers, support staff) over three additional site visits.

In summary, prior to any adaptation work, we gained extensive knowledge of both MCCAN (original EBI and context), the new context (rural Iowa hospitals), and recipients of the adapted EBI (I-CAN affiliates).

### MODIFI step 2

#### Adapt the EBI

##### Sampling and data collection

In preparation for adaptation, we created a core function/form matrix [18, 19], mapping MCCAN forms to the universe of identified intervention and implementation core functions (Table 1). We inserted blank columns to document contextual differences as well as potential adapted forms to address the contextual differences. An implementation scientist investigator and experienced qualitative interviewer then conducted a focus group to solicit our (I-CAN team’s) perspectives on potential forms for each MCCAN core function adapted to accommodate features of Iowa’s context, as well as potential barriers to operationalizing core functions in forms that would be acceptable, appropriate, and feasible for the recipients of the adapted EBI (I-CAN affiliates). This involved eliciting our knowledge of the differences between Iowa and Kentucky contexts. The focus group was conducted over the course of two, hour-long sessions via Zoom, and a research assistant took detailed field notes.

**Table 1:** Matrix of MCCAN forms mapped to its intervention and implementation core functions*

Intervention core functions	Sample forms	Contextual differences	Potential adapted forms to address contextual differences
Providing expertise about CoC standards (what they are, how they are interpreted by the CoC)	Documentation resources, training materials		
Providing a plan and framework for becoming accredited	Template for 3-year CoC accreditation timeline and Gantt chart, attendance at CoC site surveys of affiliate hospitals		
Establishing a culture of data-driven quality improvement	Facilitate sharing Quality Improvement project ideas across network hospitals		
Prioritizing the role of Oncology Data Specialist in using data to drive program enhancements	Empowerment, support and guidance for the Oncology Data Specialist to provide their affiliate hospital with quality data		
Establishing a shared goal of providing the best care for patients	Developing and maintaining strong interpersonal relationships between MCCAN and affiliate hospitals		
Providing ongoing education to assist providers and other staff to remain current and gain new skills/knowledge	Webinars, continuing education credits, educational materials, and shadowing opportunities		
Helping patients feel secure in their choice to seek care locally	Affiliates are able to use of the UK/Markey logo on buildings and for marketing		
Allowing patients to access programs and specialized services not locally available	Telemedicine with Markey for genetic counseling, affiliate liaison to help patients navigate specialized care at Markey		
Implementation core functions	Sample forms	Contextual differences	Potential adapted forms to address contextual differences
Efficient communication and access to MCCAN leaders	“First phone call,” regular meetings with MCCAN liaison		
Providing guidance and support for community outreach efforts	Marketing resources, ideas for community outreach events		
Efficient recruitment of local patients into clinical trials	Communicating open clinical trials at Markey, established process to screen and refer appropriate patients for trial		
Treatment of affiliates as equals and valued colleagues in a partnership	Open-door policy, building on affiliate hospital's program strengths, attendance at CoC site surveys of affiliate hospitals		
Trust (from affiliates) in the quality of care provided by Markey	Developing and maintaining strong interpersonal relationships between Markey and affiliate hospitals		
MCCAN is invested/enthusiastic about and supports affiliate goals while building confidence in affiliates to achieve those goals	Provide tangible support and information to help programs achieve their own goals and priorities		
Engaging providers and administrators to garner their essential support for the network	Provide education about accreditation, define specific and meaningful roles for key stakeholders		
Facilitating networking between affiliates, fostering a sense of community/kinship	Annual meeting and round table conferences with all affiliates, connecting affiliates with shared goals and challenges		
Efficient reciprocal referral process between Markey and affiliates	Affiliate liaison at Markey to help patients navigate their care while being referred, commitment to send patients back home for local treatment and follow-up		
Providing support for staff planning and recruitment	Providing job descriptions and responsibility documents to fill new roles, networking with staff counterparts at Markey to discuss roles		

*Abbreviation*: *CoC* Commission on Cancer

^*^Blank columns are designed to be completed during adaptation process

##### Data analysis

We summarized field notes from the focus groups within the function/form matrix. Each of the original EBI’s core functions (rows) had corresponding columns to document the forms implemented in the original context, known differences between the original and new contexts, and potential forms for the adapted EBI based on group consensus. We also identified additional clarifying questions to ask I-CAN affiliates (recipients of adapted EBI) in planned future interviews (MODIFI Step 3).

### MODIFI step 3

#### Evaluation of proposed adapted forms by new local users

##### Sampling and recruitment

We engaged administrators and physician partners from I-CAN affiliate hospitals (recipients of the adapted EBI, new local users) in individual semi-structured interviews designed to identify the set of forms they viewed as valuable, implementable, and potentially effective in Iowa hospitals to be included in final the adapted protocol. Specific individuals were selected based on established relationships built across multiple site visits (see MODIFI Step 1 above). To recruit at least one administrator and clinician from each site, we contacted eligible participants up to three times and offered a $50 check to incentivize participation. After initial contact, a research coordinator emailed participants to schedule the interview. Our final sample included ten participants (5 administrators and 5 clinicians). One participant was an administrator at two of affiliate hospitals and answered interview questions from the perspective of both facilities.

##### Data collection

We developed an interview guide based on the core function/form matrix (Table 1; described in MODIFI Step 2 above) to direct conversation through each core function’s potential forms for I-CAN. We emailed the function/form matrix to participants prior to interviews. After explaining a core function (column 1) and presenting the forms implemented in MCCAN (column 2), we asked participants to describe their context (column 3) and evaluate the fit of the potential forms (column 4) for their hospital. All interviews were conducted by two I-CAN team members between October 2021 and August 2022 via Zoom, audio recorded, and transcribed verbatim. One interview was not able to be recorded due to technical difficulties. However, a research assistant took detailed field notes.

##### Data analysis

A subset of the I-CAN team reviewed the results of Iowa affiliate interviews. We summarized interview responses for forms of each core function for each participant and then analyzed across participants into common themes. We identified ideal forms fulfilling affiliate preferences (e.g., in a hypothetical scenario with unlimited resources) and noted boundaries based on our knowledge of the Iowa context and resources available to carry out the EBI. We presented final adapted forms to the full I-CAN team, based on the ideal forms possible within the identified boundaries, for final review and input.

#### Ongoing adaptation and evaluation of intervention forms

As a cancer care network established to support community hospitals through the process of pursuing CoC accreditation, I-CAN by design incorporated ongoing, real-time evaluation of network activities and progress. We operationalized this evaluation at two levels: (1) between the I-CAN team and affiliate hospitals and (2) within the I-CAN team. (1) After the site visit and gap analysis meetings, I-CAN team members prepared and presented to key partners their findings and recommendations on interventions to meet each CoC standard along with a template of a three-year plan to achieve CoC accreditation. We incorporated input from the key affiliate partners (e.g., cancer center goals, priorities and strengths) into the final three-year plan, which was agreed upon by all parties. We assessed progress in meeting accreditation standards in each subsequent meeting and amended the three-year plan as needed. (2) In addition to the detailed notes taken at each meeting with affiliate hospitals, the implementation team reflected as a group after each meeting. We created a matrix for each affiliate hospital, with CoC standards listed as individual rows and columns to document the team’s observations, reflection and assessment of the affiliate hospitals’ progress and effectiveness of the I-CAN forms in achieving intended outcomes. We noted barriers and facilitators associated with specific standards, as well as ideas for next steps and recommendations to convey to the affiliates. Additional columns in the matrix listed task assignments and/or point persons. The final column documented decisions made regarding the standard and/or form.

## Results

### MODIFI step 1

#### Forms and core functions of the original EBI

We reported MCCAN forms and core functions elsewhere [23]. Briefly, we identified 18 core functions: eight intervention core functions and 10 implementation core functions. The intervention core functions mapped to effectiveness outcomes and highlighted the balance between the benefits of MCCAN affiliation (e.g., access to genetic counseling) and the dependence on MCCAN that affiliation required (e.g., relying on MCCAN expertise while pursuing CoC accreditation). The implementation core functions mapped to implementation outcomes and the Capability, Opportunity, Motivation-Behavior (COM-B) model for change [27], providing affiliates the capability, opportunity, and motivation required to achieve CoC standards.

#### Key similarities between the original and new EBI and context that support implementation

Kentucky and Iowa have longstanding contracts with NCI’s Surveillance Epidemiology and End Results (SEER) program to collect and report cancer incidence and survival data for their largely rural state-wide catchment area. Both states house a single NCI-designated cancer center: University of Kentucky Markey Cancer Center and University of Iowa Holden Comprehensive Cancer Center, which could serve as the hub of clinical services and resources for the networks. Individuals on both MCCAN and I-CAN teams held leadership roles at the state or national level within CoC.

#### Key differences between the original and new EBI and context that impact adaptation

##### Network affiliation, infrastructure, and cancer registry data collection

MCCAN was an established network with existing resources and infrastructure and housed organizationally within the Markey Cancer Center. Clinical services were provided by Markey and facilitated through a robust referral system. Although members of the I-CAN team held appointments and leadership roles within the Holden Comprehensive Cancer Center, there currently exists no official relationship between the network and cancer center. The I-CAN team, funded by a federal grant, established and resourced I-CAN, the first cancer care network in the state. There were few interactions between members of the I-CAN team and rural affiliate hospitals prior to the grant and no formal referral system existed between any of the rural hospitals and the Holden Comprehensive Cancer Center. Collection of hospital-level cancer registry data differed between the two contexts. While cancer is a reportable disease in all 50 states, Kentucky hospitals have been required to collect and report their cancer data to the state registry using a specific software application that is compliant with both SEER and CoC requirements for data submission. Up until recently, the Iowa Cancer Registry assisted hospitals that did not have their own internal cancer registry with data collection using a software application that was compliant with SEER requirements but not CoC requirements.

##### Valuation of network affiliation and readiness for quality improvement and CoC accreditation

Most hospitals approached MCCAN with an interest to join the network and perceived co-branding with the University of Kentucky Markey Cancer Center and CoC as a signal of quality and valuable benefit of membership [24]. Affiliates were required to obtain CoC-accreditation within 3 years of joining MCCAN, and all 19 community hospitals affiliated with MCCAN at the time of this writing were CoC-accredited [28, 29]. In contrast, hospitals were invited to join I-CAN as part of a funded research study, with a stated objective of supporting rural hospitals in pursuing CoC accreditation. None of the I-CAN affiliates had been CoC-accredited, and none was seriously considering pursuing accreditation when first approached. Unlike MCCAN affiliates, I-CAN affiliates were not offered co-branding with the University of Iowa Holden Comprehensive Cancer Center at the outset of the project, and when asked about the appeal of doing so expressed mixed views on the value of co-branding with the University of Iowa, partly due to other health system affiliations or proximity to other large cancer centers.

### MODIFI step 2

#### Adapt the EBI

At the end of this process, we determined that the forms from seven core functions did not require adaptation. These forms were activities driven solely by the EBI implementation team (i.e., required no effort from I-CAN affiliates) and feasible for the I-CAN team to implement with existing and/or planned resources.

Forms from a subset of 11 core functions required adaptation to the Iowa context. The contextual differences and adaptation challenges identified during this process informed the scope and nature of the potential forms. We also determined that new processes and infrastructure were needed to successfully implement I-CAN. The most significant and pressing process was to collect cancer registry data, a CoC standard that required affiliate sites to evaluate their cancer care delivery. Cancer registry data collection was not generally required of MCCAN to establish because of Kentucky state code and Kentucky Cancer Registry policies. New software technology and contracts with the Iowa Cancer Registry were required of the affiliates and additional workload on the I-CAN team to meet this accreditation standard.

We also found it difficult to adapt forms for certain core functions. Barriers to adaptation were driven by contextual differences and constraints (e.g., payment/billing differences in telehealth reimbursement, existing referral patterns, available resources and personnel). For example, “efficient reciprocal referral process between Markey and affiliates” is one of MCCAN’s implementation core functions. As one of its forms, MCCAN employed a dedicated Affiliate Liaison to facilitate incoming referrals, with duties ranging from coordinating scheduling of appointments to obtaining medical records and assisting with parking. The University of Iowa Holden Comprehensive Cancer Center used a centralized scheduling system that did not allow for an Affiliate Liaison type position to circumvent or layer on top of this system. Hospital-level changes (e.g., mergers, loss of providers, switching electronic health records vendors, changes to upper administration, etc.) that occurred over time also impacted adaptation and implementation.

### MODIFI step 3

I-CAN affiliates were generally unfamiliar with the structure and processes required for CoC accreditation, making it difficult for the I-CAN affiliates to evaluate the potential forms presented to them. I-CAN affiliates perceived the potential forms to be valuable, implementable, and potentially effective in the abstract; however, the potential forms presented to them during interviews evolved as they gained practical experience in applying the forms in collaboration with the research team. For example, providing mentorship to affiliate Oncology Data Specialists (ODS), formerly known as Certified Tumor registrars, on data analysis for quality improvement changed to using the Iowa Cancer Registry staff to perform these services for the affiliates because affiliate hospitals lacked the resources to train these data specialists in a timely fashion.

In practice, the research team used the core functions as a framework for selecting possible forms identified by I-CAN affiliate interview participants (e.g., developing a virtual ‘collaborative care consult’ service to address the need for access to subspecialty expertise across long travel distances), based on their gained knowledge of both the original EBI and context and the new context. Although I-CAN itself is the intervention, the final set of intervention forms implemented at the affiliate hospitals reflected each individual local context (i.e., hospital priorities, goals, strengths, and constraints).

## Discussion

We successfully adapted MCCAN, a complex, multilevel EBI designed to support community hospitals and enhance access to high-quality cancer services and programs in Kentucky to improve care for patients in Iowa affected by cancer—nearly half of whom reside in rural areas. The adapted EBI, I-CAN, is currently being implemented in 5 rural Iowa hospitals. All I-CAN affiliates have made significant progress towards compliance with the CoC standards, including establishing a cancer program leadership group (cancer committee), instituting a tumor board, and developing its supportive services such as rehabilitation, survivorship and palliative care. Awareness of our network has spread over time and rural hospitals are now starting to approach our team with interest in joining I-CAN, as is currently the case with MCCAN.

In contrast to many EBIs whose adaptation has been described in the literature (e.g., trauma-focused cognitive behavioral therapy [19]), MCCAN does not directly improve care or care delivery, but rather is intended to support cancer hospitals in improving care delivery and pursuing CoC accreditation. Thus, the scope of our adaptation efforts extended beyond the patients, caregivers, and providers whose perspectives MODIFI seeks to also include hospital and health system administrators. Adapting other complex, multilevel EBIs may require similarly more expansive approaches. Further, in many published adaptation efforts, recipients of the EBI in new contexts had established (yet suboptimal) processes that the EBI sought to improve (e.g., referring patients to hospice) [16]. In contrast, I-CAN affiliates lacked established processes related to the EBI. Thus, rather than engaging I-CAN affiliates in co-designing adapted forms as prescribed by MODIFI, the investigator team had to present potential forms to I-CAN affiliates, which our nuanced understanding of MCCAN, its original context, and the new Iowa context made possible.

Regardless of whether all primary users participated in the co-design of adapting forms, our approach required (1) identifying the full universe of the original EBI’s forms, (2) mapping forms to the full universe of core functions, (3) translating concrete forms of the original EBI associated with a particular core function into abstract concepts rooted in an EBI theory of change (i.e., of how the EBI changed outcomes) that transcended context and finally (4) translating the abstract EBI theory of change into concrete forms that fit the new context and fulfill the same core function in the adapted EBI. Thus, our approach to adaptation was more conceptual and dynamic in nature than a more mechanistic approach of changing the EBI’s adaptable periphery at a single time point characterized by methods like MODIFI. This “concrete forms à core functions/abstract theory of change à concrete forms” approach to adaptation is consistent with the approach described in MADI, on which MODIFI was based. We did not gather empirical data on the cost of our more conceptual, dynamic approach to adaptation; however, our adaptation efforts were funded by a large R01 and conducted by a large team of researchers, suggesting that the cost of adaptation was significant. Similarly, we have not yet generated evidence of I-CAN’s sustained use in practice, as implementation is ongoing. Future research should quantify the cost of EBI adaptation and report on adapted EBIs’ sustainability.

To our knowledge, this study was the first to apply MODIFI to an empirical study, aside from the case study featured in the paper in which MODIFI was introduced. In addition to the “concrete forms à core functions/abstract theory of change à concrete forms” approach described above, our adaptation effort suggests two other amendments to MODIFI to better align it with MADI, on which MODIFI was based.

First, MODIFI identifies three components of Step 1 without specifying an order of operations. MADI specifies that understanding of context (and users) should directly relate to aspects that are relevant to EBI core functions. That is, MADI recommends that users include those who are responsible for carrying out EBI core functions, and features of context that support EBI core functions (i.e., implementation core functions) [23]. Thus, collecting data regarding context and users must be directly informed by (and thus subsequent to articulating) EBI core functions. Indeed, scholars have developed several methods for EBI adaptation [30], most of which emphasized the importance of adapting EBIs by first identifying EBI core functions to ensure their continued effectiveness in forms identified for new contexts [19]. For example, the Map of the Adaptation Process (MAP) model instructs, “To maintain fidelity to the core [functions] identified by the original researcher, agencies should consider seeking [technical assistance] from experts in the EBI to explore the internal logic of the intervention” [31]. Research-Tested Intervention Program Adaptation Guidelines suggest that those adapting EBI should “work with expert advisors to ensure that the adapted products maintain the accuracy of the originals” [32]. Despite most adaptation methods’ recommendations to begin EBI adaptation by identifying core functions, to our knowledge, these methods (including MODIFI) neither articulate methods for doing so nor explicitly anchor adaptations on previously identified core functions. Further, previous studies have not distinguished between intervention and implementation core functions. Outcomes and theories of change are fundamentally different between these two types of core functions; failure to identify which forms map to intervention versus implementation core functions could impact adaptation and subsequent implementation.

Second, MODIFI suggests understanding the local context, i.e., the new context, to identify aspects that may interfere with intervention implementation. MADI suggests that adaptation requires specifying differences between original and new contexts to be addressed with adapted forms of EBI core functions; this was true in our experience. When possible, we retained the form(s) chosen by MCCAN for a given core function, as the original EBI has been shown to be effective. Adaptation of forms was necessary because of differences in the Kentucky and Iowa context (e.g., relationships between hospitals and processes within hospitals). Thus, in this study, we developed nuanced understanding of both original and new contexts and recommend that MODIFI be amended accordingly. MODIFI also recommends an anthropological approach to learning the users and context, one focused on identifying problems: unmet needs, interference, and poor fit. These problems are not necessarily tied to specific forms, at least not as MODIFI is articulated. When adapting forms, MODIFI focuses on generating possible solutions to these previously identified problems. Although a core function/form matrix is created in Step 1, MODIFI does not appear to recommend the matrix’s use in Step 2. If the proposed adaptation, co-designed to address an identified problem, is not mapped back to the core function of the original form, the new form may not improve the intervention or implementation outcomes the core function addresses. Our approach grounded the adapted forms to the original forms, ensuring that adapted forms not only mapped to the same original core function but that all core functions were represented in the final forms (adapted or not).

Our approach to adapting MCCAN will be of particular interest to large, decentralized networks of healthcare organizations, such as the National Cancer Institute Community Oncology Research Program, and the increasing number of horizontally integrated health systems and hospital networks that seek to scale up complex, multilevel EBIs in diverse contexts. In their efforts to scale up EBIs, healthcare organization systems and networks should anticipate and prepare for the time and financial cost associated with rigorous core functions-based EBI adaptation. Application of these methods may be best supported with existing infrastructure. For example, having identified MCCAN core functions, other adaptations could be supported through National Cancer Institute Comprehensive Cancer Centers, whose community outreach and engagement mandates ostensibly include engaging rural cancer hospitals in their catchment areas in improvement initiatives like MCCAN. The National Institutes of Health’s adaptation educational events may also be leveraged to support adaptation in practice [33].

Limitations to our method should be considered in future applications of core functions-based EBI adaptation. As the first manuscript of which we are aware to apply MODIFI, we lack evidence regarding the extent to which our process is uniquely intensive or complex, and whether our process would be equally effective in adapting other complex, multilevel interventions (i.e., whether our process is generalizable). However, we adhered to principles of MODIFI (the changes that we made to enhance feasibility of applying MODIFI as described above notwithstanding) and understand its principles to be transferable. We used MODIFI to report our adaptation of a single EBI; necessary modifications will likely depend on specific features of other EBIs to be adapted and the contexts for which they are adapted. As we have, future work should document modifications to MODIFI and their rationale. I-CAN activities were driven by core functions but varied across hospitals, limiting the relevance of a single adapted protocol. Instead, we have rigorously documented forms throughout I-CAN implementation to be reported for application in future iterations of I-CAN (i.e., a menu of forms that can be used to fulfill core functions). At that time, we will use MADI to code adaptations’ influences on implementation and intervention outcomes. To the extent that participants’ perspectives differed from others in their hospitals, our findings may be biased by the perspectives of the individuals included in interviews. The lack of engagement with CoC standards and the accreditation process limited their ability to evaluate proposed adapted forms. However, the composition, knowledge, and experience of the I-CAN team and their shared role in both adaptation and implementation minimizes the impact of this lack of familiarity on the effectiveness and implementation of the adaptation. Trained implementation scientists guided the application of adaptation methods in the context of a federally funded grant. How well these core functions-based adaptation methods can be applied without implementation science expertise, outside the context of funded research, remains unclear. The implementation and effectiveness of I-CAN, the EBI adapted using core functions-based adaptation methods, has yet to be determined but will be assessed in ongoing studies.

## Conclusions

In this paper, we reported our adaptation of a complex, multilevel EBI designed to support community hospitals and enhance access to high-quality cancer services and programs in Kentucky to improve care for patients in rural Iowa using MODIFI, representing the first empirical application of the approach of which we are aware. Applying MODIFI to a complex, multilevel EBI allowed us to identify several opportunities for refining MODIFI, including identifying EBI core functions prior to collecting context and user data, anchoring adaptations on differences between original and new contexts, and mapping adapted forms onto core functions. Future adaptation efforts may be enhanced with these refinements.

## Supplementary Information

Supplementary Material 1.

## Data Availability

The datasets used and/or analyzed during the current study are available from the corresponding author on reasonable request.

## Abbreviations

MCCAN: Markey Cancer Center Affiliate Network
CoC: Commission on Cancer
EBI: Evidence-based intervention
NCI: National Cancer Institute
I-CAN: Iowa Cancer Affiliate Network
MADI: Model for Adaptation Design and Impact
MODIFI: Making Optimal Decisions for Intervention Flexibility during Implementation
COM-B: Capability, Opportunity, Motivation-Behavior
SEER: Surveillance Epidemiology and End Results
MAP: Map of the Adaptation Process
